# Association of Platelet‐to‐White Blood Cell Ratio With All‐Cause and Cardiovascular Mortality in Hypertensive Patients

**DOI:** 10.1155/ijhy/2913197

**Published:** 2026-11-01

**Authors:** Yuqin Xiong, Han Qian, Qimei Zong

**Affiliations:** ^1^ The First Affiliated Hospital of Xiamen University Xiamen Fujian China xmu.edu.cn; ^2^ Department of Cardiology The First People’s Hospital of Taicang Taicang Affiliated Hospital of Soochow University Taicang Jiangsu China suda.edu.cn

**Keywords:** all-cause mortality, cardiovascular mortality, hypertension, nonlinear relationship, platelet-to-white blood cell ratio

## Abstract

**Introduction:**

This study investigates the prognostic value of the platelet‐to‐white blood cell ratio (PWR), a composite inflammatory indicator derived from routine complete blood count parameters, for all‐cause and cardiovascular mortality in hypertensive populations.

**Methods:**

A retrospective cohort analysis was conducted using data from the National Health and Nutrition Examination Survey (NHANES) database spanning 1999–2018. The study included 21,608 hypertensive participants aged ≥ 20 years with complete PWR measurements and mortality follow‐up data. Cox proportional hazards regression models were employed to assess associations between PWR and mortality. Restricted cubic splines identified nonlinear relationships, and time‐dependent receiver operating characteristic (ROC) curves evaluated predictive performance.

**Results:**

An inverse association was observed between PWR and mortality risk in hypertensive patients. Each standard deviation increase in PWR was associated with a 10.0% reduction in all‐cause mortality (HR = 0.900, 95% CI: 0.873–0.927, *p* < 0.001) and a 12.3% reduction in cardiovascular mortality (HR = 0.877, 95% CI: 0.828–0.930, *p* < 0.001) in fully adjusted models. A characteristic L‐shaped nonlinear relationship was identified, with inflection points at PWR 45.22 for all‐cause mortality and PWR 50.59 for cardiovascular mortality. Time‐dependent ROC analysis also demonstrated modest discriminatory capacity.

**Conclusion:**

This study suggests that PWR might be a potential biomarker for stratifying risk and predicting mortality in hypertensive patients.

## 1. Introduction

Hypertension (HTN) remains a major global health burden and a significant contributor to cardiovascular disease (CVD) morbidity and mortality [1]. HTN control continues to be a significant public health challenge [2]. Over the past 30 years, the number of adults aged 30–79 with HTN worldwide has doubled, increasing from 648 million to 1.278 billion [3]. In the United States, the prevalence of HTN among adults is approximately 45% [4]. Despite advances in treatment strategies, a substantial proportion of hypertensive patients still experience adverse outcomes, highlighting the need for reliable prognostic biomarkers to guide risk stratification and early intervention [2].

In hypertensive patients, higher systemic inflammatory burden is associated with increased risks of all‐cause and cardiovascular mortality [5]. In recent years, hematological inflammatory indices derived from routine complete blood count (CBC) parameters have gained increasing attention due to their accessibility, cost‐effectiveness, and ability to reflect systemic inflammation [5, 6]. Among these, the platelet‐to‐white blood cell ratio (PWR) has received considerable attention as an important hematological indicator that comprehensively reflects inflammatory dynamics and long‐term prognosis [7]. Studies have shown that patients with favorable recovery typically exhibit specific combined trajectories of platelets and white blood cells (WBC), which PWR effectively summarizes as an inflammatory state [7]. Previous research has demonstrated that PWR is closely associated with various chronic diseases such as diabetes mellitus (DM), sarcopenia, chronic kidney disease, and CVDs [8–12]. In various acute conditions such as acute heart failure, myocardial infarction (MI), and stroke, PWR also shows significant associations with mortality, and its predictive performance surpasses other traditional blood cell count indices and ratios [7, 13–15]. However, the specific role of PWR in HTN‐related mortality risk remains insufficiently explored, with limited data elucidating its utility in this high‐risk cohort.

The present investigation evaluates the prognostic value of the PWR for long‐term all‐cause and cardiovascular mortality in hypertensive populations using the National Health and Nutrition Examination Survey (NHANES) data. Our hypothesis posits that PWR, as a composite inflammatory indicator, serves as an independent predictor of mortality and enhances the discriminatory capacity of current prognostic models.

## 2. Materials and Methodology

### 2.1. Participant Selection

Data were extracted from the publicly available NHANES database spanning 1999–2018, with an initial sample of 101,316 participants. NHANES employs a complex, multistage probability sampling design to obtain nationally representative estimates of health and nutritional status in the United States. The study was approved by the institutional review board of the National Center for Health Statistics (https://www.cdc.gov/nchs/nhanes/about/erb.html). Exclusion criteria included (1) missing PWR measurements, (2) not meeting HTN criteria, (3) age < 20 years, and (4) unavailable mortality follow‐up data. HTN was operationalized as systolic blood pressure ≥ 140 mmHg, diastolic blood pressure ≥ 90 mmHg, physician diagnosed HTN, or antihypertensive medication use. After applying exclusion criteria, the final study population consisted of 21,608 participants.

### 2.2. PWR Calculation and Mortality Outcomes

PWR was defined as the quotient of platelet count (expressed as 10^9^/L) and WBC count (10^9^/L). Mortality information was sourced from the NHANES Public‐Use Linked Mortality Files, which are connected to the National Death Index with follow‐up through December 31, 2019. In our analysis, determination of the underlying cause of death utilized the UCOD_LEADING variable, while endpoints comprised all‐cause and cardiovascular mortality in patients with HTN (available at: https://www.cdc.gov/nchs/data-linkage/mortality-public.htm). The analytical cohort in our study exhibited a median follow‐up period of 95 months.

### 2.3. Covariates

The covariates incorporated in this analysis comprised demographic, lifestyle, clinical, and comorbidity variables. Demographic factors included age, sex, race/ethnicity, marital status, educational attainment, and poverty‐to‐income ratio (PIR). Race/ethnicity was categorized as Mexican American, Non‐Hispanic White, Non‐Hispanic Black, Other Hispanic, and Other Races. Marital status was dichotomized into married or unmarried. Educational level was grouped as less than high school and above high school. Lifestyle variables included smoking status (never smokers vs. smokers [former/current]) and alcohol use (≥ 12 drinks/year). Clinical parameters encompassed body mass index (BMI, kg/m^2^), albumin (g/L), triglycerides (TG, mg/dL), total cholesterol (TC, mg/dL), high‐density lipoprotein cholesterol (HDL‐c, mg/dL), low‐density lipoprotein cholesterol (LDL‐c, mg/dL), serum creatinine (Scr, μmol/L), and serum uric acid (SUA, mg/dL). Comorbidities included DM and CVDs. DM was defined by self‐reported diagnosis, use of insulin/oral hypoglycemic agents, hemoglobin A1c ≥ 6.5%, or fasting plasma glucose ≥ 126 mg/dL. CVDs comprised self‐reported history of MI, stroke, congestive heart failure (CHF), coronary heart disease (CHD), or angina. Detailed variable definitions are available in the NHANES documentation.

### 2.4. Statistical Analysis

Analyses of NHANES 1999–2018 data accounted for the complex multistage sampling design using R’s survey package, incorporating 2‐year mobile examination center examination weights (WTMEC2YR), primary sampling units (SDMVPSU), and strata (SDMVSTRA). Continuous data are presented as weighted means with 95% confidence intervals (CIs), and categorical variables as unweighted frequencies with weighted percentages. Missing covariates (Supporting Information: Table S1) were assumed to be missing at random and imputed using the iterative random forest algorithm implemented in the missForest R package (ntree = 100, maxiter = 10), with convergence evaluated by out‐of‐bag imputation error. Kruskal–Wallis and Rao–Scott chi‐square tests were employed for group comparisons of continuous and categorical variables, respectively. Four Cox models assessed the impact of PWR on mortality, incrementally adjusting for confounders. As this was a retrospective observational cohort study and PWR was measured only at baseline, all analyses were designed to evaluate statistical associations rather than causality. The proportional hazards assumption was evaluated using Schoenfeld residuals, with no evidence of violation detected. To evaluate the potential influence of unmeasured confounding, E‐values were calculated for the primary hazard ratios (HRs). Additionally, sensitivity analyses were performed by excluding participants with extreme PWR values. Restricted cubic splines were utilized to visualize and test for nonlinearity; the most predictive threshold was identified by maximizing likelihood, serving as the basis for two‐segment Cox modeling. Effect modification was explored in stratified analyses, and time‐dependent receiver operating curve (ROC) evaluated the dynamic predictive ability of PWR, neutrophil‐to‐lymphocyte ratio (NLR), and platelet‐to‐lymphocyte ratio (PLR) for survival outcomes. Incremental predictive performance was assessed, with the incremental value of PWR quantified using the net reclassification improvement (NRI) and integrated discrimination improvement (IDI). Analyses used R, with significance set at *p* < 0.05.

## 3. Results

### 3.1. Baseline Characteristics of the Study Cohort

The baseline characteristics of the study cohort are presented in Table 1. A total of 21,608 hypertensive participants were included in the analysis, with 15,883 classified as survivors and 5725 as nonsurvivors. Demographic comparisons indicated that nonsurvivors were characterized by older age, lower educational attainment, and reduced marital status (*p* < 0.001). Higher comorbidity rates were observed among nonsurvivors, including DM, CVDs, and smoking history, while alcohol consumption was less frequently reported (*p* < 0.001). Clinical parameter assessment revealed that nonsurvivors had lower BMI, reduced albumin levels, and decreased PIR (*p* < 0.001). Mixed lipid profile patterns were noted, with nonsurvivors demonstrating lower TG, TC, and LDL‐c concentrations but elevated HDL‐c (*p* < 0.05). Renal function markers showed greater deterioration in nonsurvivors, as evidenced by increased Scr and SUA levels (*p* < 0.001). Additionally, the PWR was found to be significantly lower in nonsurvivors (*p* < 0.001). On the other hand, as shown in Supporting Information: Figure S1, both all‐cause and cardiovascular mortality exhibited a dose‐response relationship with PWR quartiles, with the highest mortality rates observed in the lowest PWR quartile (Q1: 30.56% for all‐cause mortality and 8.73% for cardiovascular mortality) and progressively decreasing rates across higher quartiles (Q4: 23.11% and 5.82%, respectively) (*p* < 0.001).

**TABLE 1:** Comparison of baseline characteristics between survivors and non‐survivors.

Characteristic	Overall (*n* = 21,608)	Survivors (*n* = 15,883)	Non‐survivors (*n* = 5725)	*p* value
Age (years)	57.71 (57.30, 58.11)	54.83 (54.44, 55.23)	69.72 (69.20, 70.25)	< 0.001
Sex, *n*%				0.580
Female	10,954 (51.03%)	8311 (51.12%)	2643 (50.66%)	
Male	10,654 (48.97%)	7572 (48.88%)	3082 (49.34%)	
Ethnicity, *n*%				< 0.001
Mexican American	3048 (5.64%)	2360 (6.20%)	688 (3.29%)	
Other Hispanic	1612 (4.31%)	1373 (4.69%)	239 (2.71%)	
Non‐Hispanic White	10,072 (70.91%)	6613 (68.94%)	3459 (79.14%)	
Non‐Hispanic Black	5237 (12.84%)	4071 (13.23%)	1166 (11.21%)	
Other Race	1639 (6.30%)	1466 (6.93%)	173 (3.66%)	
Above high school, *n*%	9730 (54.50%)	7752 (57.76%)	1978 (40.87%)	< 0.001
Married, *n*%	11,681 (58.77%)	8905 (60.88%)	2776 (49.92%)	< 0.001
Smoking history, *n*%	10,846 (50.55%)	7499 (48.43%)	3347 (59.43%)	< 0.001
Alcohol use, *n*%	14,963 (74.34%)	11,321 (76.77%)	3642 (64.20%)	< 0.001
DM, *n*%	6477 (25.00%)	4416 (22.79%)	2061 (34.26%)	< 0.001
CVDs, *n*%	4788 (19.18%)	2558 (14.41%)	2230 (39.15%)	< 0.001
PIR	2.94 (2.89, 2.99)	3.06 (3.01, 3.12)	2.42 (2.35, 2.49)	< 0.001
BMI (kg/m^2^)	30.80 (30.64, 30.95)	31.12 (30.94, 31.30)	29.43 (29.20, 29.67)	< 0.001
Albumin (g/L)	42.06 (41.96, 42.16)	42.27 (42.16, 42.38)	41.21 (41.07, 41.35)	< 0.001
TG (mg/dL)	170.65 (167.50, 173.81)	172.15 (168.37, 175.94)	164.37 (160.34, 168.41)	0.007
TC (mg/dL)	196.85 (195.79, 197.91)	197.47 (196.25, 198.68)	194.26 (192.67, 195.85)	0.001
HDL (mg/dL)	52.36 (51.98, 52.73)	52.16 (51.74, 52.58)	53.20 (52.42, 53.97)	0.019
LDL (mg/dL)	114.51 (113.80, 115.23)	115.59 (114.78, 116.39)	110.01 (108.86, 111.16)	< 0.001
Scr (μmol/L)	84.42 (83.64, 85.20)	81.01 (80.34, 81.67)	98.69 (96.34, 101.03)	< 0.001
SUA (mg/dL)	5.77 (5.74, 5.80)	5.71 (5.68, 5.75)	6.00 (5.94, 6.06)	< 0.001
PWR	35.57 (35.23, 35.90)	35.90 (35.52, 36.27)	34.20 (33.79, 34.60)	< 0.001

*Note:* Weighted means (95% CI) for continuous variables and unweighted counts with weighted percentages for categorical variables. Group comparisons used Kruskal‐Wallis and Rao‐Scott chi‐square tests.

### 3.2. Cox Regression Analysis of PWR and Mortality in HTN Patients

Cox proportional hazards regression analysis also revealed a significant inverse association between PWR and mortality risk among hypertensive individuals (Table 2). In unadjusted models, each standard deviation (SD) increase in PWR was associated with reduced risks of all‐cause mortality (HR = 0.769, 95% CI: 0.748–0.792; *p* < 0.001) and cardiovascular mortality (HR = 0.731, 95% CI: 0.691–0.773; *p* < 0.001). After sequential adjustment for demographic, socioeconomic, and clinical covariates, these associations remained statistically significant, albeit attenuated (*p* < 0.001). In the fully adjusted model, each SD increment in PWR corresponded to a 10.0% reduction in all‐cause mortality risk (HR = 0.900, 95% CI: 0.873–0.927; *p* < 0.001) and a 12.3% reduction in cardiovascular mortality risk (HR = 0.877, 95% CI: 0.828–0.930; *p* < 0.001). The E‐values for all‐cause and cardiovascular mortality were 1.46 and 1.54, respectively, with corresponding confidence‐limit E‐values of 1.37 and 1.36. Quartile‐based analysis demonstrated a dose–response relationship, with the highest PWR quartile exhibiting 27.3% and 32.2% lower risks for all‐cause and cardiovascular mortality, respectively, compared to the lowest quartile (*p* for trend < 0.001). Sensitivity analyses yielded results materially consistent with those of the primary analyses. After excluding participants with platelet (150–400 10^9^/L) or WBC counts (4–10 10^9^/L) outside the normal range, the inverse associations of PWR with all‐cause and cardiovascular mortality remained statistically significant (Supporting Information: Table S2).

**TABLE 2:** Cox proportional hazards regression analysis of PWR and mortality risk.

PWR	Events, *n*/*N*	Model 1	Model 2	Model 3	Model 4
		HR (95% CI) *p* value			
All‐cause mortality					
Per SD increase	5725/21,608	0.769 (0.748, 0.792) < 0.001	0.839 (0.814, 0.864) < 0.001	0.866 (0.840, 0.892) < 0.001	0.900 (0.873, 0.927) < 0.001
Quantiles					
Q1	1648/5393	Ref.	Ref.	Ref.	Ref.
Q2	1459/5405	0.760 (0.708, 0.815) < 0.001	0.791 (0.737, 0.849) < 0.001	0.821 (0.765, 0.881) < 0.001	0.860 (0.800, 0.923) < 0.001
Q3	1368/5401	0.637 (0.593, 0.685) < 0.001	0.702 (0.652, 0.755) < 0.001	0.748 (0.696, 0.805) < 0.001	0.806 (0.749, 0.868) < 0.001
Q4	1250/5409	0.507 (0.471, 0.546) < 0.001	0.616 (0.571, 0.666) < 0.001	0.666 (0.617, 0.720) < 0.001	0.727 (0.672, 0.787) < 0.001
*p* for trend		< 0.001	< 0.001	< 0.001	< 0.001
Cardiovascular mortality					
Per SD increase	1605/21,608	0.731 (0.691, 0.773) < 0.001	0.799 (0.754, 0.846) < 0.001	0.823 (0.777, 0.872) < 0.001	0.877 (0.828, 0.930) < 0.001
Quantiles					
Q1	471/5393	Ref.	Ref.	Ref.	Ref.
Q2	432/5405	0.788 (0.692, 0.898) < 0.001	0.824 (0.722, 0.939) 0.004	0.850 (0.745, 0.969) 0.015	0.907 (0.795, 1.036) 0.150
Q3	387/5401	0.633 (0.553, 0.724) < 0.001	0.700 (0.610, 0.803) < 0.001	0.741 (0.646, 0.850) < 0.001	0.833 (0.725, 0.958) 0.010
Q4	315/5409	0.449 (0.389, 0.519) < 0.001	0.549 (0.473, 0.637) < 0.001	0.588 (0.506, 0.683) < 0.001	0.678 (0.582, 0.791) < 0.001
*p* for trend		< 0.001	< 0.001	< 0.001	< 0.001

*Note:* Model1: Non‐adjusted. Model 2: adjusted for age, sex, and ethnicity/race. Model 3: adjusted for age, sex, ethnicity/race, PIR, marital status, education level, smoking history, and alcohol use. Model 4: adjusted for age, sex, ethnicity/race, marital status, education level, PIR, smoking history, alcohol use, DM, CVDs, BMI, albumin, TG, TC, Scr, and SUA.

### 3.3. L‐Shaped Relationship of PWR With Mortality Outcomes

The study identified a characteristic L‐shaped nonlinear inverse association between PWR and mortality risk in hypertensive patients. Restricted cubic spline analysis depicted an initial sharp decline in mortality probability with increasing PWR, subsequently reaching a plateau (*p*‐overall< 0.001, *p*‐nonlinear< 0.05) (Figure 1). Segmented regression determined specific inflection points: PWR 45.22 for all‐cause mortality and PWR 50.59 for cardiovascular mortality (Table 3). Within the lower PWR range, each SD increase was linked to substantial mortality risk reduction. However, beyond these thresholds, the protective effect weakened.

**FIGURE 1:**
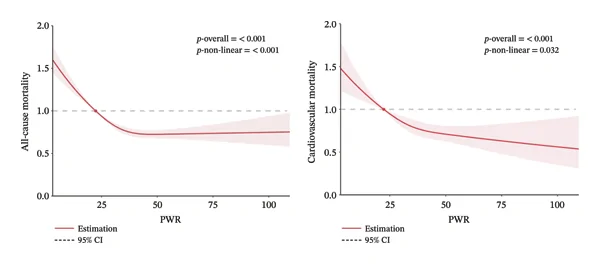
Dose‐response association of PWR with all‐cause and cardiovascular mortality (restricted cubic spline analysis).

**TABLE 3:** Threshold effects of PWR on mortality outcomes identified by segmented regression.

PWR	HR (95% CI) *p* value
All‐cause mortality	
Inflection point	45.22
PWR < 45.22	0.821 (0.787, 0.855) < 0.001
PWR ≥ 45.22	1.081 (1.017, 1.148) 0.012
*p* for Log‐likelihood ratio	< 0.001
Cardiovascular mortality	
Inflection point	50.59
PWR < 50.59	0.821 (0.765, 0.881) < 0.001
PWR ≥ 50.59	1.116 (0.964, 1.292) 0.143
*p* for Log‐likelihood ratio	0.002

*Note:* adjusted for age, sex, ethnicity/race, marital status, education level, PIR, smoking history, alcohol use, DM, CVDs, BMI, albumin, TG, TC, Scr, and SUA.

### 3.4. Stratified Analyses and Diagnostic Performance

Stratified analyses consistently revealed protective associations between elevated PWR and reduced mortality across all examined subgroups (Figure 2). No significant interaction effects were detected for age, sex, race, BMI, DM status, or history of CVDs (all *p* for interaction > 0.05). Time‐dependent ROC analysis demonstrated modest discriminatory capacity of PWR for mortality prediction throughout the follow‐up periods (Supporting Information: Figure S2). For all‐cause mortality, area under the curve (AUC) values were 0.598 at 60 months, 0.617 at 120 months, and 0.606 at 180 months. Corresponding AUC values for cardiovascular mortality were 0.608, 0.626, and 0.619, respectively. Based on Model 3, the addition of PWR yielded an NRI of 0.115 and an IDI of 0.127 for all‐cause mortality. For cardiovascular mortality, the corresponding NRI and IDI values were 0.195 and 0.082, respectively (Supporting Information: Table S3).

**FIGURE 2:**
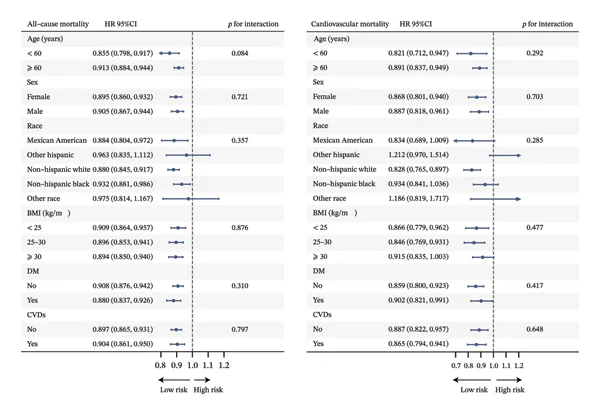
Subgroup analyses of the association between PWR and mortality.

## 4. Discussion

Using the NHANES population cohort, this study evaluated the prognostic significance of PWR for all‐cause and cardiovascular mortality in patients with HTN. The analysis identified a significant L‐shaped nonlinear inverse association between baseline PWR levels and mortality outcomes, with turning points observed. These results indicate that PWR may possess clinical utility for prognostic evaluation in HTN populations.

In comparison with biomarkers that are either more expensive or technically demanding, PWR eliminates the need for additional testing costs and specialized platforms, thus offering a practical solution for widespread adoption in primary care. The biological rationale for PWR lies in the complementary information provided by its two components. An increased WBC count may reflect systemic inflammation, whereas platelet counts may capture aspects of vascular injury, hemostatic balance, and platelet–leukocyte interaction. Accordingly, a low PWR may identify hypertensive individuals with a greater inflammatory burden and impaired vascular homeostasis. However, PWR is a composite peripheral blood marker and should not be interpreted as a direct measure of any single inflammatory or hemostatic pathway [16–18]. In this context, the L‐shaped association observed in our study is biologically plausible. Restricted cubic spline analysis showed that the inverse association between PWR and mortality gradually attenuated and approached a plateau at higher PWR percentiles, suggesting a potential saturation effect. This pattern may indicate that low PWR identifies a subgroup with disproportionately high inflammatory and vascular risk, whereas further increases in PWR provide limited additional prognostic information. Moreover, an exceptionally high PWR may reflect leukopenia, bone marrow dysfunction, medication effects, or underlying chronic disease rather than a more favorable inflammatory state. A similar nonlinear association has been reported in patients with acute decompensated heart failure [13] although the underlying mechanisms remain uncertain and warrant prospective investigation. On the other hand, given the wide CIs in the high percentile range and the potentially limited sample size of extreme values, the plateau or even mild upward trend should also be interpreted with caution. Other platelet‐derived ratios (such as platelet‐albumin ratio) have also shown associations with poor outcomes [19–21]. PWR’s advantages include straightforward calculation and minimal influence from nutritional status. Additionally, unlike PLR or NLR which have been more extensively studied, PWR encompasses the complete leukocyte population, potentially offering a more holistic assessment of systemic inflammation [22, 23]. Compared to individual platelet indices like mean platelet volume (MPV), PWR‐based nature helps offset laboratory measurement variations. To sum up, although PWR is readily calculated from routinely available CBC parameters, its clinical utility remains uncertain. The observed AUC values were modest, and further studies are warranted to evaluate whether incorporating PWR into established risk‐prediction models. Currently, PWR should not be regarded as a stand‐alone risk marker but rather as a potential complementary indicator alongside other established risk factors.

Several limitations warrant consideration in interpreting our findings. First, PWR was measured only once at baseline, whereas the follow‐up period was relatively prolonged. Changes in infections, medication use, and comorbidities may have altered PWR over time, leading to exposure misclassification and potentially attenuating the observed associations. Second, several covariates, including albumin, Scr, SUA, and lipid parameters, may lie downstream of the systemic inflammatory state captured by PWR and may partly mediate its association with mortality. Their inclusion may have resulted in overadjustment and attenuation of the total association (Supporting Information: Figure S3). Third, residual and unmeasured confounding cannot be excluded. Factors such as frailty, healthcare access, medication adherence, and antihypertensive treatment intensity may have influenced both PWR and mortality. Additionally, HTN was defined using the traditional threshold of ≥ 140/90 mmHg rather than the ≥ 130/80 mmHg threshold recommended by the 2017 American College of Cardiology/American Heart Association guidelines, which may have influenced the composition and generalizability of the study population. Finally, the stratified and quartile‐based analyses were exploratory and involved multiple subgroup evaluations and pairwise comparisons without formal adjustment for multiplicity.

## 5. Conclusion

Our findings demonstrate that PWR is associated with all‐cause and cardiovascular mortality among hypertensive participants. However, its discriminatory performance was modest, and its incremental predictive value beyond established risk factors requires further confirmation. PWR may serve as a potentially useful, low‐cost supporting marker for risk stratification, but it should not currently be considered a stand‐alone clinical prediction tool.

## Funding

The authors have nothing to report.

## Ethics Statement

The study adhered to the Declaration of Helsinki. NHANES data were used under approval by the National Center for Health Statistics Ethics Review Board (https://www.cdc.gov/nchs/nhanes/about/erb.html).

## Conflicts of Interest

The authors declare no conflicts of interest.

## Supporting Information

Additional supporting information can be found online in the Supporting Information section.

## Supporting information


**Supporting Information** Table S1: Missing covariates. Table S2: Sensitivity analysis excluding participants with extreme PWR values. Table S3: Incremental predictive value of PWR, NLR, and PLR for all‐cause and cardiovascular mortality. Figure S1: All‐cause and cardiovascular mortality across PWR quartiles. Figure S2: Time‐dependent ROC curves of PWR, NLR, and PLR for predicting all‐cause and cardiovascular mortality at 60, 120, and 180 months. Figure S3: Directed acyclic graph of covariates for the association between PWR and mortality.

## Data Availability

The data that support the findings of this study are openly available in National Health and Nutrition Examination Survey at https://wwwn.cdc.gov/nchs/nhanes.
