# Supplementary material for: Association of Platelet‐to‐White Blood Cell Ratio With All‐Cause and Cardiovascular Mortality in Hypertensive Patients
Source: Int J Hypertens. 2026 Nov 1;2026:2913197. doi: 10.1155/ijhy/2913197 (PMC13725027; doi:10.1155/ijhy/2913197)
Supplement: Supplementary file 1 [file IJHY-2026-2913197-s001.docx]

**Supplementary Material**

| **Table S1 Missing covariates.** | | |
| --- | --- | --- |
| Missing Covariates | Missing, n | Missing, % |
| Marital status | 187 | 0.87% |
| Education level | 36 | 0.17% |
| Smoking history | 20 | 0.09% |
| Alcohol use | 1459 | 6.75% |
| DM | 330 | 1.53% |
| CVDs | 234 | 1.08% |
| PIR | 1959 | 9.07% |
| BMI | 533 | 2.47% |
| Albumin | 460 | 2.13% |
| TG | 472 | 2.18% |
| TC | 395 | 1.83% |
| HDL | 395 | 1.83% |
| LDL | 4171 | 19.30% |
| Scr | 461 | 2.13% |
| SUA | 469 | 2.17% |

| **Table S2 Sensitivity analysis excluding participants with extreme PWR values.** | | | | |
| --- | --- | --- | --- | --- |
| PWR | Model 1 | Model 2 | Model 3 | Model 4 |
|  | HR (95%CI) P value | | | |
| All-cause mortality |  |  |  |  |
| Per SD increase | 0.771 (0.747, 0.796) <0.001 | 0.860 (0.832, 0.889) <0.001 | 0.880 (0.852, 0.910) <0.001 | 0.901 (0.871, 0.932) <0.001 |
| Quantiles |  |  |  |  |
| Q1 | Ref. | Ref. | Ref. | Ref. |
| Q2 | 0.816 (0.754, 0.883) <0.001 | 0.896 (0.828, 0.971) 0.007 | 0.912 (0.842, 0.988) 0.024 | 0.932 (0.861, 1.010) 0.086 |
| Q3 | 0.673 (0.621, 0.731) <0.001 | 0.812 (0.747, 0.882) <0.001 | 0.842 (0.775, 0.915) <0.001 | 0.881 (0.811, 0.958) 0.003 |
| Q4 | 0.520 (0.478, 0.566) <0.001 | 0.685 (0.627, 0.747) <0.001 | 0.721 (0.660, 0.787) <0.001 | 0.761 (0.696, 0.832) <0.001 |
| P for tend | <0.001 | <0.001 | <0.001 | <0.001 |
| Cardiovascular mortality |  |  |  |  |
| Per SD increase | 0.750 (0.706, 0.797) <0.001 | 0.842 (0.790, 0.896) <0.001 | 0.859 (0.807, 0.915) <0.001 | 0.901 (0.845, 0.960) 0.001 |
| Quantiles |  |  |  |  |
| Q1 | Ref. | Ref. | Ref. | Ref. |
| Q2 | 0.857 (0.739, 0.993) 0.040 | 0.945 (0.815, 1.097) 0.458 | 0.953 (0.821, 1.105) 0.523 | 1.002 (0.863, 1.163) 0.981 |
| Q3 | 0.684 (0.587, 0.797) <0.001 | 0.832 (0.712, 0.972) 0.021 | 0.857 (0.733, 1.001) 0.052 | 0.936 (0.800, 1.095) 0.409 |
| Q4 | 0.481 (0.409, 0.566) <0.001 | 0.640 (0.541, 0.758) <0.001 | 0.669 (0.564, 0.792) <0.001 | 0.748 (0.630, 0.889) 0.001 |
| P for tend | <0.001 | <0.001 | <0.001 | <0.001 |
| **Note:** Model1: Non-adjusted. Model 2: adjusted for age, sex, and ethnicity/race. Model 3: adjusted for age, sex, ethnicity/race, PIR, marital status, education level, smoking history, and alcohol use. Model 4: adjusted for age, sex, ethnicity/race, marital status, education level, PIR, smoking history, alcohol use, DM, CVDs, BMI, albumin, TG, TC, Scr, and SUA. | | | | |

| **Table S3 Incremental predictive value of PWR, NLR, and PLR for all-cause and cardiovascular mortality.** | | | | | | |
| --- | --- | --- | --- | --- | --- | --- |
|  |  | NRI | |  | IDI | |
|  |  | 95%CI | P value |  | 95%CI | P value |
| All-cause mortality |  |  |  |  |  |  |
| Basic model |  | Ref. |  |  | Ref. |  |
| Basic model + PLR |  | 0.017 (-0.012 - 0.046) | 0.248 |  | 0.003 (-0.023 - 0.041) | 0.922 |
| Basic model + NLR |  | 0.089 (0.058 - 0.119) | <0.001 |  | 0.162 (0.134 - 0.192) | <0.001 |
| Basic model + PWR |  | 0.115 (0.087 - 0.143) | <0.001 |  | 0.195 (0.133 - 0.263) | <0.001 |
| Cardiovascular mortality |  |  |  |  |  |  |
| Basic model |  | Ref. |  |  | Ref. |  |
| Basic model + PLR |  | 0.020 (-0.031 - 0.069) | 0.434 |  | -0.030 (-0.052 - -0.009) | 0.006 |
| Basic model + NLR |  | 0.070 (0.021 - 0.119) | 0.002 |  | 0.130 (0.080 - 0.184) | <0.001 |
| Basic model + PWR |  | 0.127 (0.080 - 0.175) | <0.001 |  | 0.082 (-0.003 - 0.161) | 0.054 |

Basic model: age, sex, ethnicity/race, PIR, marital status, education level, smoking history, and alcohol use

**Figure S1 All-cause and cardiovascular mortality across PWR quartiles.**

**Figure S2 Time-dependent ROC curves of PWR, NLR, and PLR for predicting all-cause and cardiovascular mortality at 60, 120, and 180 months.**

**Figure S3** **Directed acyclic graph of covariates for the association between PWR and mortality.**
